# The predictive factors for lymph node metastasis in early gastric cancer: A clinical study

**DOI:** 10.12669/pjms.316.8247

**Published:** 2015

**Authors:** Yinzhong Wang

**Affiliations:** 1 Yinzhong Wang, MD. Department of General surgery, The Second People’s Hospital of Jiaozuo, Jiaozuo, Henan province, China

**Keywords:** Early gastric cancer (EGC), Lymph node metastases, Depth of invasion, Local ulceration, Histological type, Venous invasion, Tumor size

## Abstract

**Objective:**

To detect the clinicopathological factors associated with lymph node metastases in early gastric cancer.

**Methods:**

We retrospectively evaluated the distribution of metastatic nodes in 198 patients with early gastric cancer treated in our hospital between May 2008 and January 2015, the clinicopathological factors including age, gender, tumor location, tumor size, macroscopic type, depth of invasion, histological type and venous invasion were studied, and the relationship between various parameters and lymph node metastases was analyzed.

**Results:**

In this study, one hundred and ninety-eight patients with early gastric cancer were included, and lymph node metastasis was detected in 28 patients. Univariate analysis revealed a close relationship between tumor size, depth of invasion, histological type, venous invasion, local ulceration and lymph node metastases. Multivariate analysis revealed that the five factors were independent risk factors for lymph node metastases.

**Conclusion:**

The clinicopathological parameters including tumor size, depth of invasion, local ulceration, histological type and venous invasion are closely correlated with lymph node metastases, should be paid high attention in early gastric cancer patients.

## INTRODUCTION

Gastric cancer, with a high prevalence and the second most common cause of cancer-related death, remains an important health problem in the world.^1^ As a result, gastric cancer has attracted high attention by the physicians. Early gastric cancer (EGC) is defined as a lesion confined to the mucosa or the submucosa, irrespective of the presence of regional lymph node metastases.^2^ Compared to advanced gastric cancer, EGC presents with a better surgical and clinical outcomes. Five-year survival rates in EGC tend to be greater than 90%,^2^ but advanced gastric cancer is usually below 5%.^3^ However, even in EGC, some patients may die from recurrence shortly after surgery. Clinical studies have showed that the prognosis of EGC with lymph node metastasis is different from those without. Lymph node metastasis (LM) is an important prognostic risk factor for EGC,^4^,^5^ following which surgeons can determine the surgical strategies.

Nowadays, many tests are available to judge the lymph node status, such as endoscopic ultrasonography and multi slice spiral computed tomography, but they are not so accurate,^6^,^7^ affecting the choice of treatment strategy adversely. When a highly invasive procedure was carried out for EGC patients without lymph node metastasis, it may results in significantly higher postoperative morbidity, mortality, reoperation rates and lower life quality.^6^ Subsequently, detecting the predictive factors for the lymph node metastasis in EGC become crucial. Some studies have been performed, but the viewpoints are different and controversial. In the study from Abe N, using multivariate logistic regression model, the authors found that female sex, a larger tumor size (20 mm or more), submucosal invasion, and presence of lymphatic vessel involvement were found to be independent risk factors for lymph node metastasis.^8^ However, Ren suggested the depth of invasion was the only independent risk factor for lymph node metastases.^2^

In this study, we retrospectively evaluated the distribution of metastatic nodes in 198 patients with EGC treated in our hospital. The objectives of our study was to detect the clinicopathological factors associated with lymph node metastases in EGC and help surgeons choose an optimal surgical treatment strategies.

## METHODS

The study cohort was composed of patients with EGC treated surgically in the department of general surgery in our hospital between May 2008 and January 2015. The inclusion criteria is the patients who (1)had a histologically confirmed gastric adenocarcinoma, (2) had a tumor with a depth confined to the mucosa or submucosa, (3) had newly diagnosed cancer without previous treatment,(4) had complete clinical and pathological data. The study has been carried out in accordance with the Declaration of Helsinki, and approved by the institutional review board of our hospital. All included patients provided written informed consent.

### Surgical procedures

It comprised distal, proximal and total gastrectomy. Proximal gastrectomy involved resection of the proximal half of the stomach, following an intraabdominal esophagogastric anastomosis. A total gastrectomy was carried out following with an esophagojejunostomy. Proximal and distal resection margins were evaluated in operation to confirm freedom from tumor. The surgical procedures and extent of lymph node dissection were based on the Japanese gastric cancer treatment guidelines.^9^

### Parameters

The relationship between various clinicopathological parameters and lymph node metastases was then analyzed. Clinicopathological parameters in the current study included age, gender, tumor location, tumor size, macroscopic type, depth of invasion, histological type, local ulceration, tumor numbers, venous invasion and so on.

The postoperative pathological examinations were carried out for all the patients, no size limitation was imposed for lymph node harvesting and each lymph node was embedded in paraffin and at least two sections were performed^2^ to detect whether the lymph nodes metastasis was available. In addition, tumor size was recorded as the maximum diameter and the size of the main tumor was measured in multiple tumors, the depth of infiltration was measured at the deepest point of penetration of cancer, tumor location was recorded as upper third, middle third or lower third gastric cancer, and the macroscopic type was classified as elevated, flat, or depressed.^9^ The above mentioned clinical factors was defined or evaluated according to the Japanese Classification of Gastric Carcinoma.^9^

### Statistical analysis

Descriptive data are recorded as the mean ± SD. The continuous variables between groups were compared using the Student’s t test, and categorical variables using χ 2 test. Parameters found to be significant (P< 0.05) in univariate analysis were included in multivariate logistic regression analysis, to identify independent risk factors associated with lymph node metastasis. The statistical analyses in the current study were carried out using SPSS 19.0 (SPSS Inc., Chicago, IL, United States), A p value less than 0.05 was considered to indicate statistical significance.

## RESULTS

One hundred and ninety-eight patients with EGC were included in this study. Of these, there were 120 men and 78 women. The age was 57.2±10.8 years, ranged from 33 to 76 years. Mucosal tumors were found in 166 patients and submucosal tumors in 32. Lymph node metastasis was detected in 28 patients, the lymph node metastases rate was 14.1%. A total of 3018 lymph nodes, including 391 from upper third, 1088 from middle third and 1539 from lower third, were removed, with a median of 15.2 lymph nodes per patient.

Univariate analysis was carried out to study the relationship between lymph node metastases and clinicopathological factors. The findings revealed a close relationship between tumor size, depth of invasion, histological type, venous invasion, local ulceration and lymph node metastases (χ 2= 4.78, P = 0.028; χ 2 = 55.74, P = 0.000; χ 2 = 7.91, P = 0.005; χ 2 = 6.15, P = 0.01, χ 2 = 5.12, P = 0.024, respectively). There was no significant correlation between lymph node metastases and gender, age, tumor location, tumor numbers, macroscopic type ((χ 2= 1.59, P = 0.21; χ 2 = 0.019, P = 0.89; χ 2 = 1.84, P = 0.17; χ 2 = 0.28, P = 0.59, χ 2 = 0.38, P = 0.53, respectively) (Table-I).

**Table-I:** Demographics of 198 patients with early gastric cancer.

Parameters	No.	LN(+) n (%)	LN(-) n (%)	X^2^	p values
*Age*					
<60 years	119	17(14.3)	102(85.7)	0.019	0.89
≥60years	79	11(13.9)	68(86.1)		
*Sex*					
Male	120	20(16.7)	100(83.3)	1.59	0.21
Female	78	8(10.3)	70(89.7)		
*Tumor location*					
Upperthird	23	2(8.7)	21(91.3)	1.84	0.17
Middlethird	64	7(10.9)	57(89.1)		
Lowerthird	111	19(17.1)	92(82.9)		
*Tumor size*					
>2cm	43	11(27.9)	32(72.1)	4.78	0.028
≤2cm	155	17(13.5)	138(86.5)		
*Tumor numbers*					
Multiple tumor	42	7(16.7)	35(83.3)	0.28	0.59
Single tumor	156	21(13.5)	135(86.5)		
*Depth of invasion*					
Mucosa	166	10(6.0)	156(94.0)	55.74	0.000
Submucosal	32	18(56.2)	14(43.8)		
*Histologic type*					
Differentiated	91	6(6.6)	85(93.4)	7.91	0.005
Nondifferentiated	107	22(20.6)	85(79.4)		
*Macroscopic type*					
Elevated	49	5(10.2)	44(89.8)	0.38	0.53
Flat	64	9(14.1)	55(85.9)		
Depressed	85	14(16.5)	71(83.5)		
*Vessel ambolus*					
+	32	9(28.1)	23(71.9)	6.15	0.01
-	166	19(11.4)	147(88.6)		
*Local ulceration*					
+	117	22(18.8)	95(81.2)	5.12	0.024
-	81	6(7.4)	75(92.6)		

LN(+):Lymph node metastasis positive; LN(-): Lymph node metastasis negative.

Multivariate analysis revealed that the depth of invasion, local ulceration, histological type, venous invasion and tumor size were independent risk factors for lymph node metastases (the OR value is 20.057, 2.895, 3.667, 2.616, 2.790 and p value is 0.000, 0.001, 0.02, 0.01, 0.024, respectively) (Table-II).

**Table-II:** Risk factors associated with lymph node metastasis.

	OR (95% CI)	P value
Tumor size	2.790(1.192-2.531)	0.024
Depth of invasion	20.057(7.781-51.700)	0.000
Histological type	3.667(1.416-9.495)	0.02
Venous invasion	2.616(1.054-6.491)	0.01
Local ulceration	2.895(1.117-7.501)	0.001

## DISCUSSION

Radical resections for gastric cancer are usually performed for EGC, but it may result in some complications including gastroplegia, reflux gastritis, inflammation of gastric relict, malnutrition and anemia, affecting the life quality of patients adversely. In recent years, some minimally invasive surgeries were carried out for EGC without lymph node metastasis, resulting in better clinical outcomes. However, this need to evaluate the status of lymph node metastasis in EGC patients. In the current study, we retrospectively evaluated the relation between metastatic nodes and clinical and pathological parameters in patients with EGC, and the study may help surgeons correctly make treatment strategies and improve the surgical outcomes of patients with EGC.

We found the depth of invasion, local ulceration, histological type, venous invasion and tumor size were independent risk factors for lymph node metastases in EGC. The depth of invasion is closely correlated with the lymph node metastasis. In the current study, the rate of lymph node metastasis was 6% in mucosa cancer, but 56.2% in submucosal cancer. There are plenty of capillary lymph ducts in submucosa and the intercellular space of endothelial cells is obvious, which result in the higher rate of lymph node metastasis when cancer occurs in submuscosa. In a study of a total of 376 patients with early gastric cancer who underwent gastrectomy, Lim found tumor size, depth of invasion, macroscopic type, and lymphovascular invasion were related to lymph node metastasis.^10^ In a study of 362 patients with early gastric cancer, Kim suggested that the presence of an ulcer is an independent predictive factor for lymph node metastasis before operation in patients with undifferentiated early gastric cancer.^11^ Shen studied retrospectively 181 patients with early gastric cancer, and concluded that histological type and tumor size is significantly and independently related to lymph node metastasis.^12^ Consequently, our results are consistent with most of the literatures.

However, in terms of the histological type, the current study revealed that it was the risk factor in predicting lymph node metastasis for the early gastric cancer patients, with an OR value of 3.667, demonstrating the incidence of lymph node metastasis is 3.667 times high in non-differentiated early gastric cancer tumors than those in differentiated tumors. However, some authors have made different conclusion. In a study of 6893 patients, Lee suggested that ulceration or non-differentiation was not associated with lymph node metastasis in elderly patients with EGC.^13^ At the same time, in a study of the comparison between 236 eldely patients and 120 young patients with EGC, Bi found that the risk factors for lymph node metastasis in young patients were different from those in elder patients. Differentiation of tumor is the risk factors in young patients, but not in elderly patients.^14^ These two studies focused on the risk factors in elder patients or the comparison between elders and youth, but the current study didn’t perform such comparative study, which resulted in the different conclusion.

As for the tumor site, we found it is not the risk factors for the lymph node metastasis. In addition, most of the gastric cancer occurred in the lower third in the current study. This clinical characteristics in the distribution of tumor site is similar with some studies,^2^,^15^ but different from others,^13^ confirming the viewpoints that the tumor site of gastric cancer is close associated with the regions.^16^

The reported rates of lymph node metastases in EGC range from 5.7% to 20%,^2^ the rate is 14.1% in this study and it is relatively lower. As a result, the reported risk factors for lymph node metastasis may be different for the reason of sample size or areas. Subsequently, we believe a large scale, multicenter clinical studies may be helpful in clarifying the issues more accurately.
